# Technological and Socio‐Demographic Factors Influencing Telemedicine Literacy in Trinidad and Tobago: A Cross‐Sectional Study Using Multidimensional Approach

**DOI:** 10.1002/hsr2.70420

**Published:** 2025-02-20

**Authors:** Ngozika E. Ezinne, Isaac Koomson, Anayochukwu E. Anyasodor, Ellen K. Antwi‐Adjei, Dipesh Bhattarai, James Armitage, Uchechukwu L. Osuagwu

**Affiliations:** ^1^ Optometry Unit, Department of Clinical Surgical Sciences University of the West Indies St. Augustine Trinidad and Tobago; ^2^ Centre for the Business and Economics of Health The University of Queensland St Lucia Queensland Australia; ^3^ Rural Health Research Institute Charles Sturt University Orange New South Wales Australia; ^4^ School of Optometry University of Alabama at Birmingham Birmingham Alabama USA; ^5^ Department of Optometry and Visual Science Kwame Nkrumah University of Science and Technology Kumasi Ghana; ^6^ School of Medicine (Optometry) Deakin University Burwood Victoria Australia; ^7^ School of Medicine, Bathurst Rural Clinical School Western Sydney University Bathurst New South Wales Australia; ^8^ African Vision Research Institute, Department of Optometry University of KwaZulu‐Natal Durban South Africa

**Keywords:** Alkire‐Foster multidimensional method, digital technology, perceived IT ability, telemedicine, telemedicine literacy, Trinidad and Tobago (T&T)

## Abstract

**Background and Aims:**

The COVID‐19 pandemic restriction impacted physical or face‐to‐face interactions, leading to an upsurge in the use of information technology (IT). This necessitated the adoption of various remote healthcare services including telehealth. This study aimed to examine the role of technological and socio‐demographic factors in enhancing telemedicine literacy.

**Methods:**

This cross‐sectional study was conducted in Trinidad and Tobago (T&T) in 2022 involving 528 participants. The study employed the Alkire‐Foster multidimensional method to measure the telemedicine literacy of participants. The multidimensional telemedicine literacy index was constructed using nine indicators spread across three dimensions (i.e., knowledge, attitude/perception, and practice dimensions), where a threshold of 0.5 was employed to identify those with adequate knowledge to be considered literate in telemedicine. The technological component was captured using IT ability. Participants completed a 31‐item questionnaire administered electronically via iPads. A “Yes” response was coded as 1 and “No” as 0.

**Results:**

Most respondents (62%) were aged 21–40, with 60% identifying as female. Most were Afro‐Trinidadian (54.46%), urban residents (84%), employed (80%), and earned a high income (87%). Overall, participants demonstrated a high perceived IT ability, with a mean score of 0.918 (SD = 0.27). Urban residents exhibited IT skills that were 10% superior to those of rural residents; however, this did not necessarily translate into higher telemedicine literacy. Gender differences were observed, with males reporting IT skills 3% higher than females. Notably, IT ability was a significant predictor of telemedicine literacy, particularly among females and urban residents. Additionally, individuals with postgraduate qualifications, Indo‐Trinidadians, and Christians exhibited significantly higher telemedicine literacy.

**Conclusions:**

This study emphasizes the pivotal role of IT ability in telemedicine literacy across varied socio‐demographic groups in T&T. To promote healthcare for all, interventions targeting digital literacy are crucial to ensure equitable access and enhance the reach of telemedicine in T&T.

## 1 Introduction

The COVID‐19 pandemic restriction impacted physical or face‐to‐face interactions, leading to an upsurge in the use of information technology [1, 2]. This necessitated the adoption of various remote healthcare services, which have been widely reported [3, 4, 5]. Telemedicine, the use of electronic communication and telecommunications technology to support long‐distance clinical service delivery [6], has received attention in recent times due to its convenience with doctors reporting high satisfaction [7]. Before the pandemic, it served as a cost‐effective alternative for people residing in remote areas with limited or deprived access to healthcare service delivery [8]. Approximately 75% of older people ( ≥ 65 years) in a multinational study in 2013 preferred booking and managing their medical conditions online [9]. On the other hand, studies showed that 37% of the world's population who are old and are on low incomes are digitally excluded and utilization of information technology reduces with an increase in age [10, 11].

The newly adopted World Health Organization (WHO) global strategy on digital health focused on supporting equitable and universal access to quality health services to make health systems more efficient and sustainable [12]. To ensure inclusive digital and equitable healthcare, WHO launched a digital app to improve care for older people in managing priority conditions including mobility limitations, malnutrition, vision and hearing loss, cognitive decline, depressive symptoms and social care and support [12].

Telemedicine involves the use of digital media, email, short messaging service (text messaging), mobile applications, interactive voice response, video conferencing, asynchronous store‐and‐forward communication, wireless communication and information technology usability or digital competency [1, 2, 6]. It has been used to promote long‐distance professional health services, disseminate medical safety reports, deliver health‐related education to the public, and carry out public health monitoring [13]. Telemedicine is useful in the control of infection during an epidemic or pandemic, including COVID‐19 [14].

A rise in computer and internet access was witnessed in several countries, including Trinidad and Tobago (T&T), coupled with the launching of telehealth [15]. In 2014, telehealth was introduced in T&T, and has delivered numerous services since its launch [4, 16]. There are indications that telehealth services will increase post‐COVID‐19 [8]. However, factors such as low computer skills, inadequate access to technology, poor internet speed or connection, cost and limited clinic resources were identified to impede telehealth use and implementation among vulnerable populations [17]. Telemedicine literacy presents challenges for various population groups such as ethnic minorities, immigrants, people with low income, the elderly and those residing in regions lacking home broadband access [18].

Digital literacy, the ability to comprehend and utilize information in diverse formats from various sources upon presentation through computers plays a critical role in influencing the effectiveness of telemedicine [19]. The skill is essential for the effective adoption and utilization of telehealth [20], as indicated in previous studies that revealed a high utilization of telemedicine among individuals with computer skills [21, 22, 23, 24, 25]. In addition, a recent study in T&T among optometrists reported poor computer skills as one of the barriers to utilization of telehealth [4]. Addressing these impediments will potentially contribute to the increased acceptance and smooth implementation of telehealth.

Telehealth utilization in the Caribbean, particularly in Trinidad and Tobago, has shown promise but remains under‐documented. It has demonstrated cost‐effectiveness and efficiency in delivering quality healthcare services. Studies indicate that telehealth has been employed in managing various health conditions, including eye, stroke, mental health disorders, diabetes, HIV, and neurological issues in Trinidad and Tobago [4, 26, 27, 28]. However, there is a significant lack of comprehensive literature on the utilization, knowledge, and perceptions of telehealth among the population across Trinidad and Tobago. A study among optometrists in Trinidad and Tobago showed that there is a high knowledge but low adoption and utilization of telehealth due to a lack of interest and concerns about the accuracy of the results obtained [4]. Also, a study in Jamaica showed that the majority prefer face‐to‐face consultation to telehealth [29]. These findings are similar to studies in Jordan, Pakistan, and Saudi Arabia [4]. Telemedicine utilization is not very common in T&T, probably due to concerns about privacy, and accuracy of the results [4]. Moreover, the fact that face‐to‐face consultation is free in public hospitals in T&T while telemedicine is not and not covered by health insurance could also be a factor.

With the increase and rapid changes in demand for telemedicine, developing and developed countries, including the USA, have designed policies to relax the hitherto tight restrictions placed on telemedicine. Some of the policies include Acts to protect the privacy and security of protected health information by healthcare providers [30]. In Europe, including France, Spain and the UK, policies that allow healthcare practitioners to conduct teleconsultation and issue e‐prescriptions were made to help relieve the burden placed on healthcare services by the pandemic. Other countries including Saudi Arabia have passed laws that allow foreign healthcare practitioners to provide telemedical services in Saudi Arabia under local practitioners' supervision [30].

Various theories have been used to describe telemedicine usability or adoption and information technology ability. For example, Rogers argues that innovations that are compatible with their environment are more easily adopted than those that are not suitable to past experiences of the adoption units [31]. However, telemedicine involves changes in patient‐provider communication, patient assessment, and engagement and acceptance. Telemedicine is a complex enterprise, involving multilevel socioeconomic and technological factors [32, 33], but how these factors influence telemedicine literacy is unclear. Koomson et al., [34].

This study contributes to the literature by examining the roles of technological and socio‐demographic factors in influencing telemedicine literacy in T&T using a novel multidimensional telemedicine literacy index that is, measured based on the Alkire‐Foster multidimensional method. Apart from the main analyses, we estimate gender‐ and location‐specific subsampled models to explore the potential heterogeneities in our findings to inform targeted telemedicine interventions. The Alkire‐Foster methodology has been used extensively across fields to construct multidimensional indexes such as poverty [35], disease outbreak resilience [34], energy poverty [36], and financial inclusion [37, 38]. Therefore, it has become imperative to apply this approach in telemedicine utilization in a local context. There is a dearth of evidence on multidimensional telemedicine literacy, and this study is the first to contribute to such conceptualization using data from T&T. Therefore, the present study aims to examine the role of technological and socio‐demographic factors in enhancing telemedicine literacy. The findings of this study will reinforce the understanding of the factors influencing telemedicine use among Trinidad population.

## 2 Methods

### 2.1 Ethical Considerations

This study adhered to the Declaration of Helsinki. Ethical approval was obtained from the Research and Ethics Committee of the University of the West Indies, Saint Augustine campus (CREC‐SA/0633/11/2020). Permissions were secured from health center administrators, and participants provided written informed consent. Participants' confidentiality was maintained, and COVID‐19 protocols were followed at the time of this study.

### 2.2 Study Design and Setting

This cross‐sectional study was conducted in Trinidad and Tobago, a twin‐island nation in the South‐eastern West Indies, encompassing a population of 1,363,985 people. For convenience and representativeness of the country, the North Central Regional Health Authority (NCRHA) was selected as the study area, since the region administers the largest number of health centers (*n* = 15) in T&T.

### 2.3 Study Population

All individuals aged 18 years and above seeking healthcare services at NCRHA health centers constituted our sampling frame. Recruitment was done through convenient sampling and spanned over 4 months (August 10 to December 30, 2022). The selection criteria included adults aged 18 years and older, those who visited the selected health centers at the time of the study, those who provided voluntary written consent. Critically ill individuals during data collection were excluded from the study.

### 2.4 Sample Size Determination

The sample size was calculated by a single‐proportion population formula, the sample size was 770, considering a confidence level of 95% (*Z*_α/2_ = 1.96), an estimated awareness and perception proportion of 50%, and a 5% margin of error. Random selection of five health centers from the 15 in NCRHA was conducted and this was followed by systematic random selection of participants in each of the selected 5 health centers, choosing every third patient from the hospital register. In cases of unavailability or unwillingness to participate, the next patient on the list was selected.

### 2.5 Data Collection

Data collection was completed by utilizing a validated self‐administered questionnaire used in previous studies [39, 40, 41, 42] and adapted for the T&T population, face validity was ensured through pretesting on 20 individuals outside the study area. The final questionnaire, consisting of 31 items, is presented in the Supporting Information S1: (S1). Research assistants and trained optometry students, collected face‐to‐face data during clinic days, ensuring accuracy through supervision and double data entry. Participants and those accompanying them to the clinics were approached by the research assistant who invited them to take part in the study. Willing participants were given the patient information sheet and the consent form. The research assistants or students were not part of the study, but they were available to assist with completing the questionnaire where necessary and on participant demand. Otherwise, the participant was given an iPad to self‐complete the survey while waiting for their appointment. Participants were informed they could ask for an explanation from the research assistants for any unclear questions. Data was entered in an Excel spreadsheet and cleaned before importing it into Stata software version 16 for statistical analysis.

### 2.6 Data Analysis

#### 2.6.1 Independent Variables

IT ability was measured as a binary variable based on the survey question, which asked respondents: “Are you able to use a computer effectively?”. A “Yes” answer was coded as 1 while a “No” answer was coded as zero. The remaining independent variables, which were derived from the items in the questionnaire, included demographics (11 items), computer use skills (3 items), presence of chronic condition (1 item), doctor interaction (1 item) and barriers to using telemedicine (1 item).

#### 2.6.2 Dependent Variable: Multidimensional Telemedicine Literacy Score

Telemedicine literacy is considered a multidimensional concept encompassing knowledge, attitude, and practice [33, 43, 44]. In this paper, we constructed a multidimensional telemedicine literacy score/index using the Alkire‐Foster approach [34, 35]. We used nine indicators of telemedicine literacy grouped under three dimensions. For the first dimension (i.e., knowledge), we employed two indicators— “knowledge of telemedicine,” and “Aware of telemedicine platforms.” For the second dimension (i.e., attitude/perception), we used six indicators that capture respondents' perceptions about telemedicine being (i) a necessity, (ii) a preferred option compared to hospital; (iii) a viable option during a pandemic; (iv) a time saver; (v) better at dealing with confidentiality issues; and (vi) option they are willing to try and use its app as used. The third dimension (i.e., practice) had one indicator—“used telemedicine before.” The survey items were from those used in previous studies [45, 46]. All indicators were captured as binary variables (Yes = 1; No = 0) to reflect adequacy in telemedicine issues.

Using the Alkire‐Foster formula as shown in Equation 1, we generated a multidimensional telemedicine literacy score by equally weighting each dimension (1/3).

(1) $${\mathrm{Tmedlit}}_{i}=w_{1}I_{1}+w_{2}I_{2}+\cdots +w_{d}I_{d},$$

where ${\mathrm{Tmedlit}}_{i}$ represents the telemedicine literacy score of respondents i. If the respondent provided an affirmative response to a particular indicator, we assigned the value 1 to indicator $I_{i}$ and 0 if otherwise. $w_{i}$ was the weight assigned to indicator I, which summed up to ${\sum_{i=1}^{d}w}_{i}=1$. A unit increase in Tmedlit indicates an improvement in telemedicine literacy. Consistent with multidimensional index construction, we were able to identify those considered literate in telemedicine issues by assigning the value 1 to individuals whose Tmedlit scores were greater or equal to 0.5 (i.e., dual cut‐off) and 0 if otherwise. From our results, the rate of multidimensional telemedicine literacy among our sampled respondents is 25.2%. Our regression analyses were done using the multidimensional telemedicine literacy score as the dependent variable. Results were presented using descriptive statistics with proportions for categorical variables and mean (with standard deviation) for continuous variables. “Yes” responses for all variables were captured as 1 and “No“ as 0. All the explanatory variables were tested based on two‐tailed tests. The analyses were all performed using STATA Version 18.0 (StataCorp LLC, College Station, TX). Reporting of the study findings followed the recommendations in the Guidelines for reporting statistics for clinical research in urology (Assel et al., 2019).

## 3 Results

### 3.1 Socio‐Demographic Profile of Respondents

Table 1 presents the demographic profile of the 528 eligible respondents in this study representing 68.6% of the desired sample size (770). The majority were within the 21‐40 age group (62%), were predominantly female (60%), resided in urban areas (84%), employed (80%), and were high‐income earners (87%). Regarding religion, the majority were Christians (77%), while in terms of education, most of the respondents had diploma certificates (47%). Considering marital status, most of the respondents were single (60%), with the remaining 40% being married. About 20% were health workers while 80% were non‐health workers. Additionally, more than half of the respondents identified as Afro‐Trinidadians (54.46%).

**Table 1:** Socio‐demographic characteristics of the study sample (*N* = 528).

Demography	Frequency	Percent
**Age group**		
< 20 years	18	3.41
21–40 years	328	62.12
41–60 years	128	24.24
> 61 years	54	10.23
**Gender**		
Male	210	40
Female	315	60
**Employed**		
No	102	19.77
Yes	414	80.23
**Health workers**		
No	420	79.55
Yes	108	20.45
**Level of education**		
Diploma	239	46.50
Bachelors	201	39.11
Postgraduate	74	14.4
**Income status**		
Low	68	12.98
High	456	87.02
**Marital status**		
Single	319	60.42
Married	209	39.58
**Religion**		
Non‐Christians	119	22.58
Christian	408	77.42
**Location**		
Urban	436	83.52
Rural	86	16.48
**Ethnicity**		
Afro‐Trinidad	287	54.46
Indo‐Trinidad	68	12.9
Mixed	172	32.64

### 3.2 Summary Statistics for Perceived IT Ability and Telemedicine Literacy by Gender and Location

**Figure A1:**
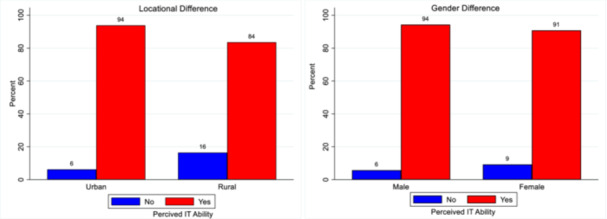
Differences in perceived IT ability by Gender (right panel) and location (left panel) in Trinidad and Tobago.

Mean scores for telemedicine literacy and IT ability are shown in Table 2, while gender and locational differences in IT ability are presented in Figure A1, which provides insight into socio‐demographic heterogeneities in IT proficiency in the surveyed population. From Table 2, it was revealed that the respondents had an overall high mean score of 0.918 (SD: 0.27) for telemedicine literacy. A significant proportion, 91.8% (*n* = 484), demonstrated high perceived IT ability, while the remaining 8.2% did not.

**Table 2:** Summary statistics showing mean scores of perceived IT ability by sociodemographic variables.

Variables	Description	Mean	SD
Telemedicine score	Multidimensional telemedicine literacy score obtained using the Alkire‐Foster method	0.341	0.243
IT ability	Binary variable equals 1 if respondent self‐reports having IT ability	0.918	0.274
**Age group**			
< 20 years	Binary variable equals 1 if respondent's age is up to 20 years	0.034	0.182
21–40 years	Binary variable equals 1 if respondent's age is from 21 to 40 years	0.621	0.486
41–60 years	Binary variable equals 1 if respondent's age is from 41 to 60 years	0.242	0.429
> 60 years	Binary variable equals 1 if respondent's age is from 61 years above	0.102	0.303
Female	Binary variable equals 1 if respondent is female	0.600	0.490
Health worker	Binary variable equals 1 if respondent is employed in the health sector	0.205	0.404
Employed (nonhealth worker)	Binary variable equals 1 if respondent is employed	0.802	0.399
Postgraduate education	Binary variable equals 1 if respondent has attained postgraduate education	0.144	0.351
High‐income status	Binary variable equals 1 if respondent is in the high‐income bracket	0.870	0.336
Married	Binary variable equals 1 if respondent is married	0.396	0.489
Christian	Binary variable equals 1 if respondent is a Christian	0.774	0.419
Rural	Binary variable equals 1 if respondent resides in the rural area	0.165	0.371
**Ethnicity**			
Afro‐Trinidad	Binary variable equals 1 if respondent's ethnicity is Afro‐Trinidad	0.545	0.498
Indo‐Trinidad	Binary variable equals 1 if respondent's ethnicity is Indo‐Trinidad	0.129	0.336
Mixed	Binary variable equals 1 if respondent's ethnicity is female Afro‐Trinidad	0.326	0.469

As shown in Figure A1, 94% of males perceive having IT ability while 91% of female consider themselves as having such skill. The gender difference in perceived IT ability is 3%, but this was nonsignificant. We also observed that 94% of urban residents perceived having IT ability while this was 84% among rural respondents. The rural‐urban difference in perceived IT ability was 10%. The statistical significance of these differences is only tested and reported in the regression analyses.

### 3.3 Regression Analysis

In this section, we applied ordinary least squares regression analysis to examine the association between perceived IT ability and multidimensional telemedicine literacy among Trinidadians and report the results in Table 3. In this model, we controlled for socio‐demographic variables such as age, gender, occupation, educational status, income, marital status, location, and ethnicity. These variables have been identified as determinants of digital health literacy in previous studies [47, 48]. We also controlled for religion because the World Health Organization often partners with religious groups and faith‐based organizations on health literacy and knowledge programs to enhance health outcomes [12]. Based on previous studies, religious affiliation is expected to be significantly associated with health behavior and health outcomes [49, 50]. Estimates from the full sample are displayed in Column 1 while the male‐ and female‐specific results are reported in Columns 2 and 3, respectively. Rural and urban subsampled estimates are respectively reported in Columns 4 and 5.

**Table 3:** Association between perceived IT ability and Telemedicine literacy (values are coefficients).

	(1)	(2)	(3)	(4)	(5)
		Gender		Location	
Variables	Full	Male	Female	Rural	Urban
**IT ability**	**0.106** ***	0.033	**0.098** **	0.103	**0.109** **
	(0.039)	(0.082)	(0.049)	(0.093)	(0.045)
**Age cohort (Base: 61 years and above)**					
Up to 20 years	0.035	0.113	0.077	−0.063	0.148
	(0.082)	(0.095)	(0.118)	(0.126)	(0.101)
21–40 years	0.045	0.031	0.086	−0.026	0.074
	(0.046)	(0.054)	(0.069)	(0.108)	(0.052)
41–60 years	0.042	−0.000	0.112	0.037	0.065
	(0.044)	(0.045)	(0.072)	(0.105)	(0.051)
**Gender**					
Female	**0.102** ***	—	—	0.030	**0.114** ***
	(0.022)	—	—	(0.075)	(0.024)
**Occupation**					
Health worker	0.035	0.010	0.043	−0.026	0.040
	(0.031)	(0.041)	(0.044)	(0.072)	(0.036)
Employed (nonhealth worker)	−0.039	−**0.074** *	−0.007	−0.007	−0.058
	(0.033)	(0.038)	(0.047)	(0.058)	(0.039)
**Education**					
Postgraduate education	**0.113** ***	**0.104** **	**0.111** **	**0.302** ***	**0.097** ***
	(0.034)	(0.041)	(0.049)	(0.089)	(0.036)
**Income**					
High‐income status	0.052	**0.109** **	0.032	−0.082	**0.078** **
	(0.034)	(0.044)	(0.045)	(0.093)	(0.036)
**Marital status**					
Married	0.002	0.004	0.000	−0.037	0.007
	(0.026)	(0.034)	(0.036)	(0.071)	(0.028)
**Religion**					
Christian	**0.074** ***	0.020	**0.105** ***	−0.015	**0.092** ***
	(0.028)	(0.036)	(0.039)	(0.059)	(0.032)
**Location**					
Rural	0.022	0.064	−0.003	—	—
	(0.029)	(0.046)	(0.040)	—	—
**Ethnicity (Base: Afro‐Trinidad)**					
Indo‐Trinidad	**0.093** **	0.044	**0.134** **	‐0.033	**0.103** **
	(0.041)	(0.057)	(0.058)	(0.092)	(0.046)
Mixed	**0.041** *	0.010	**0.066** *	0.017	0.042
	(0.024)	(0.033)	(0.035)	(0.067)	(0.027)
Constant	0.013	0.138	0.035	**0.316** *	−0.046
	(0.067)	(0.123)	(0.077)	(0.185)	(0.069)
Observations	449	175	274	68	381
*R* ^2^	0.116	0.111	0.103	0.213	0.133

*Note:* Robust standard errors in parentheses. Bolded are significant variables. For the model in Columns 2 & 3, these were segregated by gender and thus gender was not included. In Columns 4 & 5, we segregate for rural‐urban, so we do not report for rural and urban in these Columns.

*** *p*< 0.01

** *p* < 0.05

* *p* < 0.1.

For the main results in Column 1, we observed that IT ability is associated with a 0.106 increase in respondents' telemedicine literacy and this relationship was significant at the 1% alpha level. For the gender dimension, we observed in Columns 2 and 3 that IT ability did not significantly influence telemedicine literacy among males, but it did for females. Among females, we observed that IT ability is linked to a 0.098 increase in multidimensional telemedicine literacy.

The locational dimension of the results in Columns 4 and 5 also revealed that IT ability had a significant impact on telemedicine literacy among urban residents, but this was not the case among rural residents where there was no relationship between IT ability and telemedicine literacy. IT ability was associated with a 0.109 increase in multidimensional telemedicine literacy among urban residents.

Gender‐wise, we found that telemedicine literacy is 0.102 higher among females than males, mainly observed in the urban areas of T&T. The increased telemedicine literacy associated with higher education is more pronounced among females and rural residents. Christians scored 0.074 higher in telemedicine literacy than non‐Christians, and this was mainly observed among females and those residing in urban areas. Considering ethnicity, Indo‐Trinidadians and Mixed Trinidadians were 0.093 and 0.041, respectively, more literate in telemedicine compared to Afro‐Trinidadians.

## 4 Discussion

This is the first study to employ a multidimensional construct to investigate the intricate relationship between perceived IT ability, socio‐demographic factors, and telemedicine literacy among Trinidadians. Nearly all respondents had a high IT ability (91.8%), with a notable concentration among urban, employed, and high‐income individuals aged 21–40 years. Similar findings were recorded in Jamaica [51] and Ecuador [52]. Other studies [53, 54] also reported similar findings. This outcome might be expected in most places due to the rapid transformation in the use of IT experienced due to COVID‐19. This is also driven by Trinidad and Tobago being at the forefront of technological advancement in the Caribbean due to their high GDP [55]. However, no similar study has been done in the Caribbean or Latin America to compare with our findings.

In the present study, we observed that IT ability was positively associated with respondents' telemedicine literacy. This implies that efforts made to enhance the IT ability of Trinidadians will enhance Telehealth utilization, thereby improving health outcomes in the process. We also found that males and urban residents reported higher perceived IT ability.

The predilection of males in IT ability recorded in the current study stems from a mix of cultural, educational, and psychological influences. Historically, IT has been portrayed as a male‐dominated field, reinforcing stereotypes that suggest technology is a masculine pursuit. This early socialization can shape interests, with boys typically encouraged to engage in technical activities more than girls. Additionally, educational experiences contribute to this gap, as girls may receive less exposure to computing and technical subjects, impacting their confidence and interest in these areas. Also, males are known to have greater access to IT and electronic devices compared to women in this geographical region [56]. This notwithstanding enhanced IT ability for men did not significantly improve their telemedicine literacy. On the contrary, a slight improvement in IT ability among women made a significant difference in driving their curiosity in exploring and seeking newer knowledge, including telemedicine resources and platforms available to improve their health [57]. This outcome was also potentially driven by the evidence that women utilize healthcare more than men and were often more driven to seek knowledge about flexible ways of receiving healthcare. Despite substantial advancements in information and communication technologies (ICTs), there are ongoing gender disparities in access and use of IT. Research on gender differences in ICT usage often presents conflicting findings. While some studies highlight that females have more IT ability than males [52], others provide varying results, reflecting a broader debate on the impacts of gender on technology access and utilization [58].

Existing literature has established a positive relationship between socioeconomic status and urban residence and enhanced IT proficiency [53, 59, 60]. This study indicated that identifying as Christian was also associated with a higher telemedicine literacy score. Several studies indicated that there may be a willingness to adopt digital or online media resources among Christian communities in T&T [61]. These findings reaffirm established trends wherein IT skills are influenced by factors such as access to resources and sociocultural considerations [54, 62]. Moreover, the prevalence of Christianity within the Trinidadian population could potentially have impacted the results.

Interestingly, our findings do not suggest a significant difference in IT ability or multidimensional telemedicine literacy between health workers and individuals employed in nonhealthcare occupations. Rather, the results of this study identified an additional dimension to the impact of IT ability on telemedicine literacy. While a high proportion of both men and women perceived themselves as IT proficient, a significant gender variation in the association between IT ability and telemedicine literacy was revealed. Although IT ability had no significant influence on the telemedicine literacy of males, it was associated with a 0.098 increase in the telemedicine literacy of females. This finding suggests that despite the generally higher IT ability for males, the incremental improvement in IT ability among women had a more substantial impact in driving their curiosity and engagement with telemedicine resources. These findings are similar to those of other studies in Israeli, US, and Canadian populations [54, 59, 63] and maybe reflective of women demonstrating a higher utilization of healthcare services in general [64, 65], which translates into motivating them to seek knowledge about flexible healthcare options. It also highlights the importance of considering diverse factors influencing telemedicine engagement for different demographic groups.

Our analysis of urban and rural residents revealed that while urban residents demonstrated higher IT ability, location did not significantly impact telemedicine literacy. Similar findings were reported in studies in China [66] and the USA [59]. This apparent disparity can be explained by the documented deprivations in education and technology among rural residents [67, 68, 69], who predominantly prefer informal healthcare facilities [70, 71]. Rural residents, despite having lower IT ability, may not be as concerned about telemedicine issues due to preferences for informal healthcare facilities [72, 73]. On the other hand, factors beyond IT ability, such as access to healthcare facilities, awareness, and satisfaction with prior use of telemedicine, may play a more substantive role in shaping telemedicine literacy among urban residents [60]. The busy work schedules, widespread access to mobile phones, and high population density in urban areas leading to longer hospital waiting times, may provide strong motivation for urban residents to engage with telemedicine resources [74, 75]. Urban life often involves juggling work, family, and other commitments, leaving little time for traditional in‐person medical appointments. Telemedicine offers a convenient alternative, allowing people to consult with healthcare providers from the comfort of their homes or offices, thus saving time and reducing the need for travel. Moreover, telemedicine can help address the shortage of primary care physicians and specialists in urban areas, making it easier for residents to access medical care without long wait times. This convenience is particularly beneficial for those who may have difficulty scheduling appointments during regular office hours due to their busy lifestyles.

## 5 Limitations and Strengths

While our study provides valuable insights, it is essential to acknowledge its limitations. The cross‐sectional nature of the data and analytical strategy limits our ability to establish causation. Additionally, self‐reported data on IT ability and telemedicine literacy may introduce response bias. Concerns about the confidentiality of information, lack of rebate or health insurance coverage for telemedicine, quality of care or accuracy of results from telemedicine, lack of interest or difficulty accepting changes, regulatory and legal barriers are some of the factors that could affect telemedicine literacy and telemedicine adoption in T&T. The focus on a single country (Trinidadians) may limit generalizability to other populations. No information about the respondents health condition was included in our study and this may have affected the findings of this study since we were unable to control for this in our regression model. However, the strengths of our study lie in the comprehensive examination of socio‐demographic variables and the nuanced analysis of gender and locational dimensions. The large sample size and use of regression analysis strengthen our findings. Future research could explore these relationships longitudinally and incorporate qualitative methods to gain deeper insights into individuals' attitudes and behaviors towards telemedicine in diverse socio‐cultural contexts.

## 6 Implications of Study Findings

The nuanced insights from our study hold significant implications for both clinical practice and public health initiatives, particularly in the context of promoting telemedicine literacy and adoption among diverse demographic groups in T&T. The identification of a high prevalence of IT ability among the sampled respondents, and especially among urban residents and males, underscores the potential for targeted telemedicine interventions among these subgroups. Healthcare providers can leverage this existing IT proficiency to enhance patient education and engagement with telemedicine resources, thereby fostering a more informed and tech‐savvy healthcare consumer base. The observed gender variation in the impact of IT ability on telemedicine literacy, with a more substantial influence among females, suggests that tailoring telemedicine education and outreach efforts to address specific gender‐related factors could be beneficial. This could involve creating targeted educational materials that resonate with the healthcare‐seeking behaviors of women who demonstrated a heightened interest in exploring and seeking knowledge about telemedicine resources. Furthermore, recognizing the lower IT ability among specific demographic groups highlights the need for targeted interventions to bridge the digital divide. Public health campaigns and educational programs should be designed to address the unique barriers faced by these groups, focusing on building foundational IT skills and increasing awareness of the benefits of telemedicine. While our study identified higher IT ability among urban residents, the lack of a significant impact on telemedicine literacy suggests that additional factors beyond IT proficiency play a crucial role in telemedicine adoption in urban settings. Public health strategies should therefore consider a multifaceted approach, addressing not only IT skills but also factors such as accessibility, awareness, and convenience to maximize the effectiveness of telemedicine initiatives in urban areas.

## 7 Conclusions

The study emphasizes the relationship between IT ability, socio‐demographic factors and telemedicine literacy. Our main finding suggests the need to address disparities in digital literacy, coupled with interventions that target demographic groups and recognizing that IT ability alone may not be the sole determinant of telemedicine literacy.

Integrating the above finding into policy design is critical to ensuring unrestricted access to telemedicine, thereby encouraging inclusive healthcare in T&T. It also aligns with the broader global efforts to create inclusive and technology‐driven healthcare solutions that cater to the diverse needs of populations. Health promotion programmes aimed at encouraging digital literacy will favor telemedicine utilization. With the continuing evolution of telemedicine, understanding these complexities is crucial for designing inclusive and effective strategies to enhance healthcare accessibility for diverse populations. Policies targeted at improving IT skills, especially among the identified groups whose proficiency requires improvement will be beneficial.

## Author Contributions

**Ngozika E. Ezinne** and **Isaac Koomson:** conceptualization, data curation, investigation, methodology, project administration, resources, software, supervision, validation, visualization, writing–original draft, writing–review and editing. **Isaac Koomson**, and **Anayochukwu E. Anyasodor:** data curation, formal analysis, investigation, methodology, software, validation, visualization, writing–original draft, writing–review and editing. **Ellen K. Antwi‐Adjei:** data curation, formal analysis, investigation, methodology, software, validation, visualization, writing–original draft, writing–review and editing. **Ngozika E. Ezinne, Dipesh Bhattarai, James Armitage**, and **Uchechukwu L. Osuagwu:** data curation, investigation, methodology, validation, visualization, writing–original draft, writing–review and editing. **Ngozika E. Ezinne, Dipesh Bhattarai, James Armitage**, and **Uchechukwu L. Osuagwu:** data curation, investigation, methodology, validation, visualization, writing–original draft, writing–review and editing. All authors approved the final version of the manuscript.

## Ethics Statement

The study adhered to the Declaration of Helsinki and was approved by the Research Ethics Committee of the University of the West Indies, Saint Augustine Campus, Trinidad and Tobago (CREC‐SA/0633/11/2020). Written informed consents were obtained from all participants before data collection. Anonymity and confidentiality of participant responses were maintained.

## Conflicts of Interest

The authors declare no conflicts of interest.

## Transparency Statement

Uchechukwu Levi Osuagwu the corresponding author has full access to all the data in this study and takes complete responsibility for the integrity of the data and affirms that this manuscript is an honest, accurate, and transparent account of the study being reported; that no important aspects of the study have been omitted; and that any discrepancies from the study as planned (and, if relevant, registered) have been explained.

## Supporting information

Supporting information.

## Data Availability

All of the individual participant data collected during the study, after deidentification, have been provided in the accompanying supplementary file.
