# Supplementary material for: Technological and Socio‐Demographic Factors Influencing Telemedicine Literacy in Trinidad and Tobago: A Cross‐Sectional Study Using Multidimensional Approach
Source: Health Sci Rep. 2025 Feb 20;8(2):e70420. doi: 10.1002/hsr2.70420 (PMC11840707; doi:10.1002/hsr2.70420)
Supplement: Supplementary file 1 — Supporting information. [file HSR2-8-e70420-s001.pdf]

Awareness, Attitude and Perception of Trinidad & Tobago population towards use of Telemedicine

You are welcome to participate in this survey about Telemedicine in Trinidad and Tobago. Please select the best options that best describes your response. The questionnaire remains anonymous and all your responses will be kept confidential. By clicking on the link below you have accepted to participate in the survey. Thank you for your time.

* Indicates required question

DEMOGRAPHIC PROFILE

1. Please indicate your gender *

Mark only one oval.

- Female
- Male
- Prefer not to say
- Other: ____________________

2. Please indicate your age *

Mark only one oval.

- less than 20
- 21 -40 years
- 41-60 years
- 61-80 years
- Above 80 years

3. Please indicate your marital status

Mark only one oval.

- Single
- Married
- Separated
- Widowed
- Divorced
- Common law marriage

4. Please indicate your religion

Mark only one oval.

- Christianity
- Islamic
- Hinduism
- Judaism
- Budhaism
- Atheism
- Traditional religion
- Others

5. Please indicate your ethnic group

Mark only one oval.

- Indo-trinidad
- Afro-trinidad
- Mixed
- Others

6. Please indicate where you live

Check all that apply.

- [ ] Urban
- [ ] Semi-urban
- [ ] Rural

7. Please indicate your level of education

Mark only one oval.

- ( ) No formal education
- ( ) Primary school
- ( ) Secondary school
- ( ) College
- ( ) University
- ( ) Others

8. Please indicate your highest level of qualification

Mark only one oval.

- ( ) Certificate
- ( ) Diploma
- ( ) Degree
- ( ) Masters
- ( ) PhD
- ( ) Doctorate

9. Please indicate your occupation below

____

10. Please indicate your employment status

Mark only one oval.

- Government employed
- Privately employed
- Both
- NGO employed
- Self employed
- Unemployed

11. Please indicate your economic status

Mark only one oval.

- Poor (Less than 3000.00 TTD (MINIMUM WAGE)
- Not so good (3000 TO 5000 TTD)
- Average/reasonable (6000 TO 10, 000 TTD)
- Good (11,000 TO 29, 000 TTD)
- Excellent (30, 000 AND ABOVE)

12. Do you have any chronic ailment

Mark only one oval.

- Yes
- No
- Maybe

Untitled Section

COMPUTER USE SKILL

13. Are you able to use a computer effectively?

Mark only one oval.

- Yes
- No
- Maybe

14. Please indicate your level on the use of computer

Mark only one oval.

- Beginner
- Average
- Professional

15. Which smart device do you use?

____

16. How often do you interact with your doctor online via email or social media?

Mark only one oval.

- Very often (More than once in a week
- Often (at least once in a week)
- Rarely (less than once in a month)
- Never

AWARENESS OF TELEMEDICINE

17. Are you aware of telemedicine?

Mark only one oval.

- Yes
- No
- Maybe

18. What is telemedicine?

Mark only one oval.

- The remote diagnosis and treatment of patients by means of telecommunication technology
- Patients using the internet to search their symptoms and conditions
- Doctors consultation over phone and text messages
- Emailing the reports of patients who cannot travel or immobile doctors

19. Are you aware of any platform used in telemedicine?

Mark only one oval.

- Yes
- No
- Maybe

20. If yes, please state the name of the platform

PERCEPTION OF TELEMEDICINE

21. Do you agree on the necessity of telemedicine

Mark only one oval.

	1	2	3	4	5	
Stro	◯	◯	◯	◯	◯	Strongly agree

22. Have you used telemedicine before

Mark only one oval.

- ◯ Yes
- ◯ No
- ◯ Maybe

23. If yes, how satisfied are you with the service

Mark only one oval.

	1	2	3	4	5	
Not	◯	◯	◯	◯	◯	well satisfied

24. Better preference to going to hospital during a pandemic

Mark only one oval.

	1	2	3	4	5	
Stro	◯	◯	◯	◯	◯	Strongly agree

25. Willingness to use telemedicine in the future

Mark only one oval.

	1	2	3	4	5	
Stro	⬭	⬭	⬭	⬭	⬭	Strongly agree

26. Telemedicine is a viable approach for providing medical care services to patients

Mark only one oval.

- ⬭ Strongly disagree
- ⬭ Disagree
- ⬭ Neutral
- ⬭ Agree
- ⬭ Strongly agree

27. Use of telemedicine system can save time and money

Mark only one oval.

- ⬭ Strongly disagree
- ⬭ Disagree
- ⬭ Neutral
- ⬭ Agree
- ⬭ Strongly agree

ATTITUDE TOWARDS USE OF TELEMEDICINE

28. Are you concerned with privacy issues or confidentiality of your information if you interact with your doctor online?

Mark only one oval.

- Yes
- No
- Maybe

29. Would you be willing to try mobile based app health care?

Mark only one oval.

- Yes
- No
- Maybe

30. Please indicate mode of telemedicine you would prefer

Check all that apply.

- [ ] Video consultation
- [ ] Audio consultation
- [ ] Text based consultation
- [ ] Combination of any of the two
- [ ] All of the above
- [ ] None of the above

BARRIERS TO USING TELEMEDICINE

31. Please tick the major barrier to using telemedicine in Trinidad and Tobago

Check all that apply.

- [ ] Not interested
- [ ] Unfamiliar with the technique
- [ ] Cost
- [ ] Policy barriers
- [ ] Privacy and security concern
- [ ] Concern with accuracy of results
- [ ] Unavailabilty of trained staff
- [ ] Poor internet
- [ ] No guideline for use
- [ ] others

Thank you very much for your time

This content is neither created nor endorsed by Google.

Google Forms
